# Identification of the Non-Volatile Taste-Active Components in Crab Sauce

**DOI:** 10.3390/foods8080324

**Published:** 2019-08-07

**Authors:** Tian-Tian Liu, Ning Xia, Qin-Zhi Wang, De-Wei Chen

**Affiliations:** Department of Food Science, Guangxi University, Nanning 530004, China

**Keywords:** crab sauce, taste-active components, taste activity value, omission test, addition test, equivalent umami concentration

## Abstract

Crab sauce is a traditional umami seasoning in the coastal cities in South East China. The putative non-volatile taste-active components in crab sauce were measured, and their impacts on the taste were evaluated on the basis of the taste activity value (TAV), omission test, addition test and equivalent umami concentration (EUC). The EUC used to evaluate the synergistic effect of the flavor nucleotides and umami amino acids was 19.3 g monosodium glutamate (MSG)/100 mL, which illuminated that crab sauce had a very intense umami taste. The key non-volatile taste-active components in crab sauce demonstrated by the omission test and addition test were glutamic acid (Glu), aspartic acid (Asp), glycine (Gly), alanine (Ala), lysine (Lys), histidine (His), 5′-inosine monophosphate (IMP), 5′-guanosine monophosphate (GMP), NaCl, KCl, serine (Ser) and valine (Val), and most of these components also had a higher TAV. So, the TAV could be a high-efficiency tool to predict the taste-active components, and the TAV combined with the omission test and addition test could be a very useful method to determine the taste-active components in crab sauce.

## 1. Introduction

Crab sauce is a traditional umami seasoning in the coastal cities in South East China. Crab sauce is made from soldier crabs by curing and fermenting for approximately 30–40 days. During curing and fermenting, crab meat is hydrolyzed to free amino acids and peptides by its own endogenous protease and microorganisms [1]. As a fermented product, crab sauce not only provides rich nutrients, but also contributes a pleasant umami taste to the food [2,3,4,5], thus it is favored by the local people and tourists.

The taste of the fermented aquatic products include umami, saltiness, sourness, sweetness and bitterness [4,5,6,7], and the umami taste is the most important taste [3,8]. Fermented aquatic products are made from fresh aquatic material which are rich in large number of umami substances, such as umami amino acids and nucleotides [3,5,9]. After fermentation, the contents of some specific umami components, such as glutamic acid increases sharply [4,10]. Some reports showed that flavor nucleotides and MSG-like amino acids could have a synergistic effect [11], and the equivalent umami concentration (EUC) was effectively used to measure this synergistic effect in lots of food matrices [9,11,12,13]. The EUC means that the umami intensity given by the mixture of monosodium glutamate (MSG)-like amino acids (glutamic acid and aspartic acid) and flavor nucleotides is equivalent to the concentration of MSG. Due to the synergistic effect, aquatic products contribute a strong umami taste and have higher EUCs [4,14].

The taste-active value (TAV) or dose over threshold (DOT) is the ratio of the concentration of the component in the food matrix to its corresponding taste recognition threshold. The TAV is a simplified method for evaluating the contribution of the taste-active components in the complicated food matrix [9,15]. When the TAV is greater than 1, the component contributes to the taste. However, the TAV does not consider the possibilities of synergistic effect or masking effect in the food matrix, so the sensory analysis methods, such as the omission test and addition test, by omitting or adding extractive component(s), succeed in assessing the taste-active components in food [6,13,16]. Glutamic acid was the most important taste-active component in fish sauce according to the omission test and addition test [17].

Fish sauce is a typical fermented aquatic product. Free amino acids were the main taste component of fish sauce [8,17], and different amino acids usually exhibit different taste profiles and their taste impacts also depend on their respective content, taste threshold, and the interaction of other components [18,19]. These amino acids widely present in different products but differed in their concentrations, which contributed to the characteristic taste of different products [16,20].

Although there are some reports about the non-volatile taste-active compounds in fish sauce [8,17], to the best of our knowledge, there is scant research concerning the characteristic taste of crab sauce. The non-volatile taste-active compounds in crab sauce could differ because of different raw materials and fermentation times. The purpose of this study was to identify the non-volatile taste-active compounds, especially the umami taste-active compounds in crab sauce by the EUC, TAV, omission test and addition test.

## 2. Materials and Methods

### 2.1. Chemicals

All the reagents used were of analytical grade and purchased from Sinopharm Chemical Reagent Co., Ltd. (Shanghai, China). All the standards were purchased from Sigma-Aldrich (Shanghai) Trading Co., Ltd. (Shanghai, China).

### 2.2. Preparation of Crab Sauce

Crab sauce was prepared according to the traditional home-made method. Fresh soldier crabs (*Mictyris brevidactylus*) (individually weighed 3–5 g, diameter 30–40 mm) were captured in Beihai, China, during their maturation period (in October 2016). They were transported to the laboratory and cleaned immediately. Then, crab meat (from claws, legs and abdomen) was mashed and mixed with 30% salt and placed in a 2-L water-sealed fermenter. Crab sauce was obtained after fermentation for 35 days under natural conditions (18–28 ℃).

### 2.3. Free Amino Acid Analysis

Free amino acids were analyzed using the method reported by Yoshikawa et al. [21]. Free amino acids were extracted in trichloroacetic acid and then separated using the automatic amino acid analyzer (L-8900, Hitachi, Tokyo, Japan). The amino acid content was expressed as milligrams per 100 mL of crab sauce. All analyses were performed in triplicate.

### 2.4. Nucleotide Analysis

The nucleotides were extracted and analyzed by a method reported by Chen et al. with slight modifications [9]. A volume of 5 mL of crab sauce was diluted with 75% methanol solution to a final volume of 50 mL, and then centrifuged at 8000 rpm for 10 min. A volume of 1 mL of supernatant was diluted to a final volume of 50 mL and then filtered through a 0.22-μm membrane. A volume of 10 μL of the sample was analyzed by HPLC. The HPLC system consisted of an Alliance model 2695 (Waters, Milford, MA, USA) coupled to a UV detector (at 254 nm) and Agilent TC-C18 column (250 × 4.6 mm, Agilent, Santa Clara, CA, USA).

### 2.5. Organic Acids Analysis

The organic acids were analyzed by a method reported by Chen et al. with slight modifications [9]. A volume of 5 mL of crab sauce was diluted with ultrapure water to a final volume of 50 mL and was filtered through a 0.22-μm membrane. Then 10 μL of the sample was analyzed by HPLC. The HPLC condition was the same as for the nucleotide analysis except that the detector wavelength was 215 nm.

### 2.6. Inorganic Ion Analysis

The inorganic ions of sodium (Na^+^) and potassium (K^+^) were measured by inductively coupled plasma optical emission spectrometry (ICP-OES) (7700e, Agilent, Santa Clara, CA, USA) after microwave-assisted digestion with HNO_3_/H_2_O_2_ [22]. Then, the contents of Na^+^ and K^+^ were converted to the contents of NaCl and KCl.

### 2.7. Taste Activity Value (TAV)

TAV was calculated according to the following formula:

TAV = The concentration of the component in the sample (mg/100 mL) / threshold value (mg/100 mL)

### 2.8. Equivalent Umami Concentration (EUC)

The EUC (g MSG/100 mL) is given by the following equation:

EUC=∑a_m_b_m_+1218(∑a_m_b_m_)(∑a_n_b_n_)

where a_m_ is the concentration (g/100 mL) of each umami amino acid (Asp or Glu); b_m_ is the relative umami concentration (RUC) for each umami amino acid to MSG (Glu, 1; Asp, 0.077); a_n_ is the concentration (g/100 mL) of each umami 5-nucleotide (5’-IMP or 5’-GMP); b_n_ is the RUC for each umami 5’-nucleotide to 5’-IMP (5’-IMP, 1; 5’-GMP, 2.3) and 1218 is a synergistic constant based on the concentration of g/100 mL used [11].

### 2.9. Taste Profile Analysis

Based on the results of the quantitative analysis of the taste substances, a complete taste recombinant was prepared, and the differences in taste between the crab sauce and the complete taste recombinant were determined through sensory evaluation. In order to prevent taste fatigue, the crab sauce and the complete taste recombinant were diluted 5 times for the sensory evaluation. The panel consisted of 10 members (6 women and 4 men) who had been trained to discriminate the different taste and intensity. The intensity evaluation was performed using a 5-point rating scale (0, no taste; 1, weak; 2, medium; 3, strong; 4, very strong), and evaluation tastes included umami, saltiness, sweetness, sourness and bitterness. All the tests were performed in duplicate in a separate session, giving a total of 20 responses.

### 2.10. Omission Test and Addition Test

The omission test and addition test were carried out on the basis of the method reported by Kani et al. and Rotzoll et al. with minor modifications [16,23]. First, each of the partial recombinants was tested in comparison with the complete taste reconstituent, using a triangle test. Ten panelists were asked to rate the intensity of the five-given taste on a scale from 0–4. The results of the omission test could determine the main taste components, and the mixture of these main taste components was called the main components recombinant.

Then, the addition test was performed. The addition test required adding a single component or a mixture (the concentration of each component was the same as that in the complete taste recombinant) to the main components recombinant, and then a sensory evaluation was performed using the triangle test. Usibng both the omission tests and addition tests, the key non-volatile taste-active components in crab sauce could be obtained.

### 2.11. Statistical Analysis

Statistical analysis was performed by SPSS 23.0 (IBM, Armonk, NY, USA), and one-way analysis of variance was used for the significant difference test (*p* < 0.05). The data were presented as the mean ± standard deviation.

## 3. Results and Discussion

Table 1 shows the contents, taste thresholds, taste attributes and TAVs of the free amino acids in crab sauce. The total amount of free amino acids in crab sauce was 3479.3 mg/100 mL (the weight of 100 mL crab sauce was 125 g), which was higher than those in the fresh crab meat (1620 mg/100 g) [14], as it was a comprehensive result of the action of the microorganisms and the endogenous protease [4]. Glu was found in the highest concentration, and then Gly, Asp, Ala and Arg. These amino acids in crab sauce were widely presented in the fermented aquatic products, such as fish sauce and shrimp paste [3,17].

According to the taste characteristics of free amino acids, they were divided into three categories: umami amino acids, sweet amino acids and bitter amino acids [18]. The content of total sweet amino acids in crab sauce was the highest, accounting for 42% of the total amino acids, followed by total umami amino acids (33%) and total bitter amino acids (25%). So, umami amino acids and sweet amino acids were the dominant taste-active amino acids, which was consistent with the results identified by the TAVs. Human perception of the taste is not only related to the content of the taste substances, but also related to the taste thresholds of the taste substances, thus the TAV is a useful tool to evaluate the taste impact [9]. The TAVs of each free amino acid in crab sauce are shown in Table 1. Among them, the TAVs of Glu, Arg, Ala, Lys, His, Asp, Gly and Met were greater than 1, and these free amino acids could have direct contributions to the taste of crab sauce.

Glu was the most important taste-active component in fermented sauce [4,25]. When Glu is combined with sodium to form MSG, it a strong umami taste and it loses its own sour taste [26]. The TAV of Glu was 25.3, so Glu was the key umami amino acid in crab sauce and had an important contribution to the umami taste of crab sauce. Asp was the second largest umami amino acid in crab sauce. Ala and Gly were representatives of sweet amino acids and the main sweetness source of invertebrates such as shellfish and crab [14,27,28]. Met is a specific bitter amino acid. However, Arg and other bitter amino acids, in certain conditions, could enhance the mellow sense of fermented products, and give a unique taste but can also enhance the umami taste [20]. The above-mentioned amino acids were also the key taste-active components in fish sauce [17], but their concentrations were different with crab sauce, which contributed to the different characteristic taste of crab sauce.

Flavor nucleotides (mainly IMP and GMP) are the characteristic sources of the umami taste in aquatic products [9,24], which have a synergistic effect with the umami amino acids, thus they can significantly improve the umami taste [11,26]. As shown in Table 1, the contents of IMP and GMP in crab sauce were 29.7 mg/100 mL and 49.1 mg/100 mL, respectively, and their TAVs were 1.2 and 3.9 respectively, so both IMP and GMP were direct contributors to the taste of crab sauce. The content of IMP in crab sauce was significantly lower than that in fresh crab meat, but the content of GMP in crab sauce was higher [14]. However, IMP and GMP were not detected in some fish sauces [29], and the differences in nucleotides might be because the fermentation time of crab sauce was more shorter than that of the fish sauce, as nucleotides would be degraded gradually during the fermentation [5].

Organic acids in fermented products mainly come from the biochemical reactions of raw materials and microorganisms [5,30], and the type and content of organic acids would directly affect the quality of aquatic fermented condiments [31]. As can be seen from the Table 1, malic acid, lactic acid, succinic acid and acetic acid were detected in crab sauce, while tartaric acid and citric acid were not detected. Usually, organic acids induce a sour taste. However, succinic acid exhibits a umami taste besides its sour taste [20,24]. Most of the TAVs of other organic acids (except succinic acid) were lower than 1, so it could be deduced that only succinic acid could have a direct effect on the taste of crab sauce, and the others could be excluded as important taste contributors.

As large quantity of salt was added into the crab sauce; therefore, it is not surprising that the TAV of NaCl was much higher than 1. The TAV of KCl was 1.6 (Table 1). Thus, it could be concluded that both NaCl and KCl could contribute a salty taste to crab sauce.

**Figure 1:**
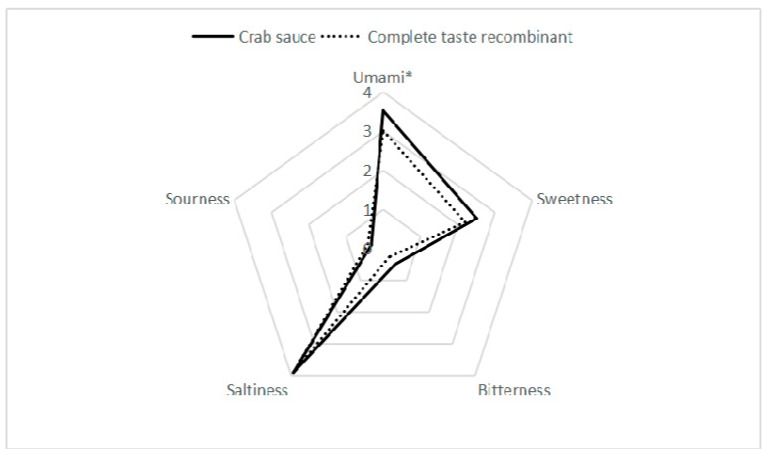
Taste profile of crab sauce (solid line) and the complete taste recombinant containing 24 compounds (dotted line). * significant difference (*p* < 0.05).

Based on the quantitative analysis of free amino acids, nucleotides, organic acids and inorganic ions, a complete taste recombinant containing the natural concentrations of the 24 compounds given in Table 1 was prepared, and the pH value was adjusted to neutral (the pH value of crab sauce was between 7–7.5) by adding some trace amounts of NaOH. Then, the taste profiles of crab sauce and the complete taste recombinant are shown in Figure 1. Crab sauce had an intense umami taste and salt taste, with a medium sweet taste, but very weak bitterness and sourness were felt. Because the synergistic effect between umami amino acids and flavor nucleotides could enhance the umami taste [11], as well as the fact that sodium chloride and certain acids could inhibit and reduce the bitter taste [32,33], the bitterness was reduced or even covered up, eventually showing the umami, salt and sweet taste characteristics.

The taste differences between crab sauce and the complete taste recombinant could be seen from Figure 1. Among the five basic tastes, except umami, there was no significant difference for the other four basic taste intensities between the samples. In addition, the taste profile of the complete taste recombinant was considered close to that of natural crab sauce and could create the typical taste of crab sauce. This result was consistent with the study by Park et al. [17], as they found that although the artificial synthetic model could reproduce the taste of fish sauce, its strength was weaker than natural fermented fish sauce. As there were other components, such as peptides present in the fermented sauce, and certain peptides can contribute or enhance the umami taste [4,34,35,36], this might explain why the artificial synthetic model had a lower umami taste intensity.

To find out the key taste compounds of crab sauce, a series of omission tests and addition tests were carried out. In the first step of the omission tests, all the putative taste-active components which comprised the complete taste recombinant (Table 1) were divided into six groups on the basis of their categories and TAVs, as shown in Table 2. Partial recombinants lacking in each of the six groups were prepared and then subjected to the sensory test using the complete taste recombinant as the reference. The results of the triangle test and the sensory changes of the omitting test are shown in Table 2. Panelists believed that the lack of group A led to the decline of sweetness and umami taste, and group A had a great influence on the taste of crab sauce; the lack of group B caused the reduction of sweetness, and the lack of group D caused the reduction of sweetness and umami taste. The absence of the low content of amino acids (group C) significantly reduced the bitterness of crab sauce. The panelists also believed that the lack of group F led to the decline of sweet, salty and umami tastes. Therefore, group A, B, C, D and F had great impacts on the crab sauce, and they would be further tested. Whereas in the absence of group E, the panelists believed there was no significant impact on the taste of reduced taste recombinant, so group E was excluded from the further omission tests.

In the next step of the omission tests, based on the results of the first omission tests and due to the higher content and TAV of each ingredient in group A, D and F, these substances were widely considered to be taste-active substances in many aquatic products and their fermented products [4,20], so each substance in these groups was removed for the omission test independently. For group B and C, they were subdivided into a single component or sub-group of the mixed components, while group E was removed. The experimental grouping and results of the second omission tests are shown in Table 3.

According to the results of the omission tests, it could be determined that Asp, Glu, Gly, Ala, Lys, His, IMP, GMP, NaCl and KCl were the taste-active components of crab sauce. The absence of Asp, Glu, Ala, IMP, GMP, as well as NaCl and KCl, caused a significant decrease in the umami taste, while the absence of Asp, Glu, Gly, Ala, Lys, IMP, GMP and NaCl could directly reduce the sweetness. The absence of His and Lys directly led to a significant reduction in bitterness, while the absence of NaCl resulted in an increase in bitterness. Saltiness was significantly reduced in the absence of NaCl and KCl. The absence of Arg, group B1, B2, C1, C2, C3 and C4 did not affect the overall taste, so these groups were excluded. Group C had an impact on crab sauce’s bitter taste and sour taste in the first omission tests. However, when it was divided into sub-groups, each sub-group had no impact on the taste, which suggested that the interactions among amino acids play important roles in imparting a bitter taste and sour taste to the crab sauce.

In the final step, 10 taste-active components (Asp, Glu, Gly, Ala, Lys, His, IMP, GMP, NaCl and KCl) determined by the omission test were prepared as the main components recombinant, and other components which were determined as non-taste-active components by the omission tests were added into the main components recombinant one by one. The results of the addition tests (Table 4) showed that Ser, Ile and Val were the taste-active substances of crab sauce.

Through the omission test and addition test, the taste-active substances in crab sauce were Asp, Glu, Gly, Ala, Lys, His, IMP, GMP, NaCl, KCl, Ser, Ile and Val. By comparing the results of the TAVs, most components except Arg, Met and succinic acid with higher TAVs had a direct impact on the tastes of crab cause, and the components except Ile with lower TAVs had no direct impact on the tastes of crab cause. So, the TAV could be a high-efficiency tool to predict the taste-active components in food, and the TAV combined with the omission test and addition test could be a powerful method to determine the taste-active components in food.

The synergistic effect of the umami amino acids and the flavor nucleotides in crab sauce was evaluated by the EUC value. The EUC value of crab sauce was calculated as 19.3 g MSG/100 mL (15.5 g MSG/100 g); that is, the umami intensity of 100 mL of crab sauce was equivalent to 19.3 g MSG. Therefore, it could be concluded that the crab sauce had a strong umami taste, as proved by the sensory analysis. Compared with previous studies [9,14], the EUC value of fresh soldier crab meat was 6.4 g MSG/100 g, so the EUC value of crab sauce was approximately 2.4-fold higher than that of fresh soldier crab meat. The main reason for this was that crab sauce contained much more umami amino acids and GMP, thus the umami taste became more intense after fermentation.

## 4. Conclusions

In short, crab sauce had a very intense umami taste with an EUC of 19.3 g MSG/100 mL. Through TAVs, omission tests and addition tests, the non-volatile taste-active components in crab sauce were determined to be Asp, Glu, Gly, Ala, Lys, His, IMP, GMP, NaCl, KCl, Ser, Ile and Val. The TAV combined with the omission test and addition test was a very useful method to determine the taste-active components in crab sauce.

## Figures and Tables

**Table 1:** The contents, taste attributes, taste threshold and TAV of the putative non-volatile taste-active components in crab sauce (mean ± SD).

Components	Taste Attribute ^1^	Threshold Value ^2^ (mg/100 mL)	Content (mg/100 mL)	TAV
Umami amino acids				
Asp	Umami and sweet (+)	100	401.8 ± 0.9	4
Glu	Umami (+)	30	758.7 ± 2.9	25.3
Total umami amino acids			1160.5 (33%)	
Sweet amino acids				
Ser	Sweet (+)	150	148.6 ± 0.7	1
Gly	Sweet (+)	130	422.9 ± 2.6	3.3
Thr	Sweet (+)	260	138.8 ± 3.3	0.5
Ala	Sweet (+)	60	340.9 ± 1.0	5.7
Lys	Sweet and bitter (−)	50	258.2 ± 0.8	5.2
Pro	Sweet and bitter (+)	300	152.8 ± 1.6	0.5
Total sweet amino acids			1462.2 (42%)	
Bitter amino acids				
Arg	Sweet and bitter (+)	50	301.3 ± 4.7	6
His	Bitter (−)	20	99.2 ± 1.7	5
Tyr	Bitter (−)	/	67.2 ± 0.4	/
Val	Sweet and bitter (+)	40	57.8 ± 2.1	1.5
Phe	Bitter (−)	90	61.4 ± 0.2	0.7
Ile	Bitter (−)	90	70.4 ± 1.5	0.8
Leu	Bitter (−)	190	142.9 ± 1.3	0.8
Met	Sweet and bitter (−)	30	56.4 ± 0.1	1.9
Total bitter amino acids			856.6 (25%)	
Total free amino acids			3479.3	
Flavor nucleotides				
IMP	Umami (+)	25	29.7 ± 0.9	1.2
GMP	Umami (+)	12.5	49.1 ± 2.2	3.9
Organic acid				
Tartaric acid	Sour and thrill (+)	1.5	ND	/
Malic acid	Sour and bitter (+)	50	13.16 ± 0.78	0.3
Lactic acid	Sour and bitter (+)	126	71.02 ± 0.21	0.6
Citric acid	Sour and mild (+)	45	ND	/
Succinic acid	Sour and umami (+)	10.6	16.17 ± 1.01	1.5
Acetic acid	Sour and thrill (+)	10.6	9.84 ± 0.52	0.9
Inorganic ion				
KCl	Salt (+)	130	205.4 ± 4.4	1.6
NaCl	Salt (+)	150	2228.9 ± 15	15.9

^1^ Taste attributes (+ = pleasant, − = unpleasant) [19,24]. ^2^ Taste threshold value (mg/100 mL) in water [18,24]. Abbreviations: Ala, alanine; Arg, arginine; Asp, aspartic acid; Glu, glutamic acid; Gly, glycine; His, histidine; Ile, isoleucine; Leu, leucine; Lys, lysine; Met, methionine; Phe, phenylalanine; Pro, proline; Ser, serine; Thr, threonine; Tyr, tyrosine; Val, valine. IMP, 5′-inosine monophosphate; GMP, 5′-guanosine monophosphate; ND, not detected.

**Table 2:** Results of the first omission tests.

Omitted Components	N^1^	Sig ^2^	Description of Taste Difference
A (Glu, Gly, Asp, Ala, Arg, Lys)	20	***	Less sweet, less umami
B (Ser, Thr, Leu, Pro, His)	17	***	Less sweet
C (Val, Met, Phe, Ile, Tyr)	15	***	Less bitter
D (GMP, IMP)	16	***	Less sweet, less umami
E (malic acid, succinic acid, lactic acid, acetic acid)	6	ns	
F (NaCl, KCl)	20	***	Less sweet, less umami, less salt

^1^ Number of correct identifications (*n* = 20) detecting a taste difference by the triangle test. ^2^ Significance: ***, very highly significant (*p* < 0.001, *n* > 14); ns, not significant (*p* > 0.05, *n* < 11).

**Table 3:** Results of the second omission tests.

Omitted Components	N^1^	Sig ^2^	Description of Taste Difference
Asp	19	***	Less sweet, less umami
Glu	20	***	Less sweet, less umami
Gly	15	***	Less sweet
Ala	15	***	Less sweet, less umami
Arg	10	ns	
Lys	15	***	Less sweet, less bitter
B1 (Thr, Pro, Leu)	9	ns	
B2 (Ser)	10	ns	
B3 (His)	14	**	Less bitter
C1 (Phe, Ile)	4	ns	
C2 (Val)	9	ns	
C3 (Tyr)	8	ns	
C4 (Met)	5	ns	
IMP	14	**	Less sweet, less umami
GMP	18	***	Less sweet, less umami
KCl	14	**	Less umami, less salt
NaCl	20	***	Less sweet, less umami, less salt, more bitter

^1^ Number of correct identifications (*n* = 20) detecting a taste difference by the triangle test. ^2^ Significance: ***, very highly significant (*p* < 0.001, *n* > 14); **, highly significant (*p* < 0.01, *n* > 13); ns, not significant (*p* > 0.05, *n* < 11).

**Table 4:** Results of the addition tests.

Added Components	N ^1^	Sig ^2^	Description of Taste Difference
Arg	10	ns	
Leu	8	ns	
Ser	16	***	More sweet, less bitter
Pro	10	ns	
Tyr	6	ns	
Ile	16	***	Less sweet, more bitter
Phe	2	ns	
Val	16	***	Less sweet, more bitter
Tyr	10	ns	
Met	10	ns	

^1^ Number of correct identifications (*n* = 20) detecting a taste difference by the triangle test. ^2^ Significance: ***, very highly significant (*p* < 0.001, *n* > 14); ns, not significant (*p* > 0.05, *n* < 11).

## Abbreviations

Ala: alanine; Arg, arginine; Asp, aspartic acid; Glu, glutamic acid; Gly, glycine; His, histidine; Ile, isoleucine; Leu, leucine; Lys, lysine; Met, methionine; Phe, phenylalanine; Pro, proline; Ser, serine; Thr, threonine; Tyr, tyrosine; Val, valine. MSG, monosodium glutamate. IMP, 5′-inosine monophosphate; GMP, 5′-guanosine monophosphate. EUC, equivalent umami concentration. TAV, taste activity value.
